# Supplementary material for: Splice-Junction-Based Mapping of Alternative Isoforms in the Human Proteome
Source: Cell Rep. Author manuscript; Available in PMC 2020 Jan 15. (PMC6961840; doi:10.1016/j.celrep.2019.11.026)
Supplement: 3 [file NIHMS1546469-supplement-3.zip › DF2/PXD0010154/Liver-149-Q9UHX1-QTIAHQQQQLTNLQMAAQR.pdf]

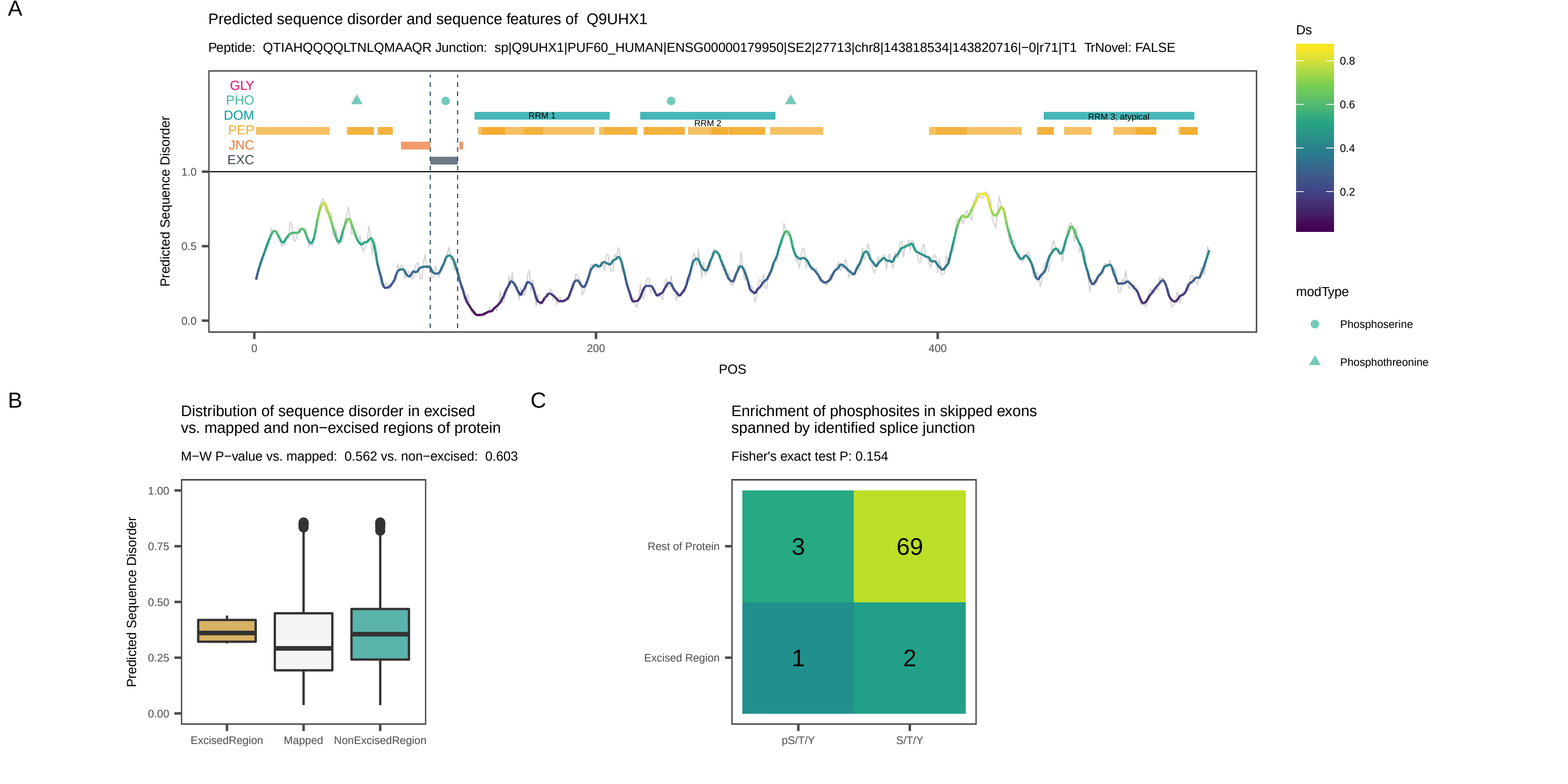
A
Predicted sequence disorder and sequence features of Q9UHX1
Peptide: QTIAHQQQQLTNLQMAAQR Junction: sp|Q9UHX1|PUF60_HUMAN|ENSG00000179950|SE2|27713|chr8|143818534|143820716|−0|r71|T1 TrNovel: FALSE
GLY
PHO
DOM
PEP
JNC
EXC
RRM 1
RRM 2
RRM 3; atypical
Predicted Sequence Disorder
1.0
0.5
0.0
0
200
400
POS
Ds
0.8
0.6
0.4
0.2
modType
Phosphoserine
Phosphothreonine
B
Distribution of sequence disorder in excised vs. mapped and non−excised regions of protein
M−W P−value vs. mapped: 0.562 vs. non−excised: 0.603
Predicted Sequence Disorder
1.00
0.75
0.50
0.25
0.00
ExcisedRegion
Mapped
NonExcisedRegion
C
Enrichment of phosphosites in skipped exons spanned by identified splice junction
Fisher's exact test P: 0.154
Rest of Protein
Excised Region
3
69
1
2
pS/T/Y
S/T/Y
